# Warming, thresholds and extinctions: Lessons from past hyperthermals on land and at sea

**DOI:** 10.1017/ext.2026.10020

**Published:** 2026-08-03

**Authors:** Pasquale Raia, Silvia Castiglione, Giorgia Girardi, Alessandro Mondanaro

**Affiliations:** DiSTAR, University of Naples Federico II, Italy

**Keywords:** global warming, mass extinctions, megafauna extinction, thermal stress, global change

## Abstract

The current biodiversity crisis is driven primarily by human activities, including global warming resulting from human greenhouse-gas emissions, which act as multifaceted environmental stressors. Rising temperatures compromise organismal performance through thermal stress, creating demographic erosion and triggering ecological cascades that propagate across populations and ecosystems. The fossil record offers a unique archive for evaluating these dynamics, documenting biological responses to a wide spectrum of past climate regimes, including warming events far exceeding those experienced in human history and the conditions projected to take place during the coming century. Drawing on events across the Phanerozoic, we emphasize mechanisms that link climate change to extinction in both marine and terrestrial realms. Extreme ancient warming episodes reveal a recurring “deadly trio” of global warming, ocean acidification and deoxygenation, processes that mirror the emerging drivers of the contemporary crisis. Focusing on the Cenozoic and the Pleistocene Ice Ages, we review mammalian responses to rapid climatic fluctuations and highlight the synergistic contribution of *Homo* to Late Quaternary megafaunal collapse. Our synthesis shows that past crises provide key insights into extinction thresholds, rates of environmental change, and the vulnerability of ecological functions under warming. Critically, comparison with ancient analogs suggests that future extinction risk on land may exceed anything observed during past warming episodes, due to the unprecedented velocity of modern warming combined with direct anthropogenic pressures such as habitat loss and fragmentation. In the oceans, projected warming and deoxygenation point toward extinction intensities comparable to those of the most extreme hyperthermal events. Collectively, the evidence indicates that Earth is approaching temperature conditions historically associated with major extinction episodes.

## Graphical abstract

**Figure:**
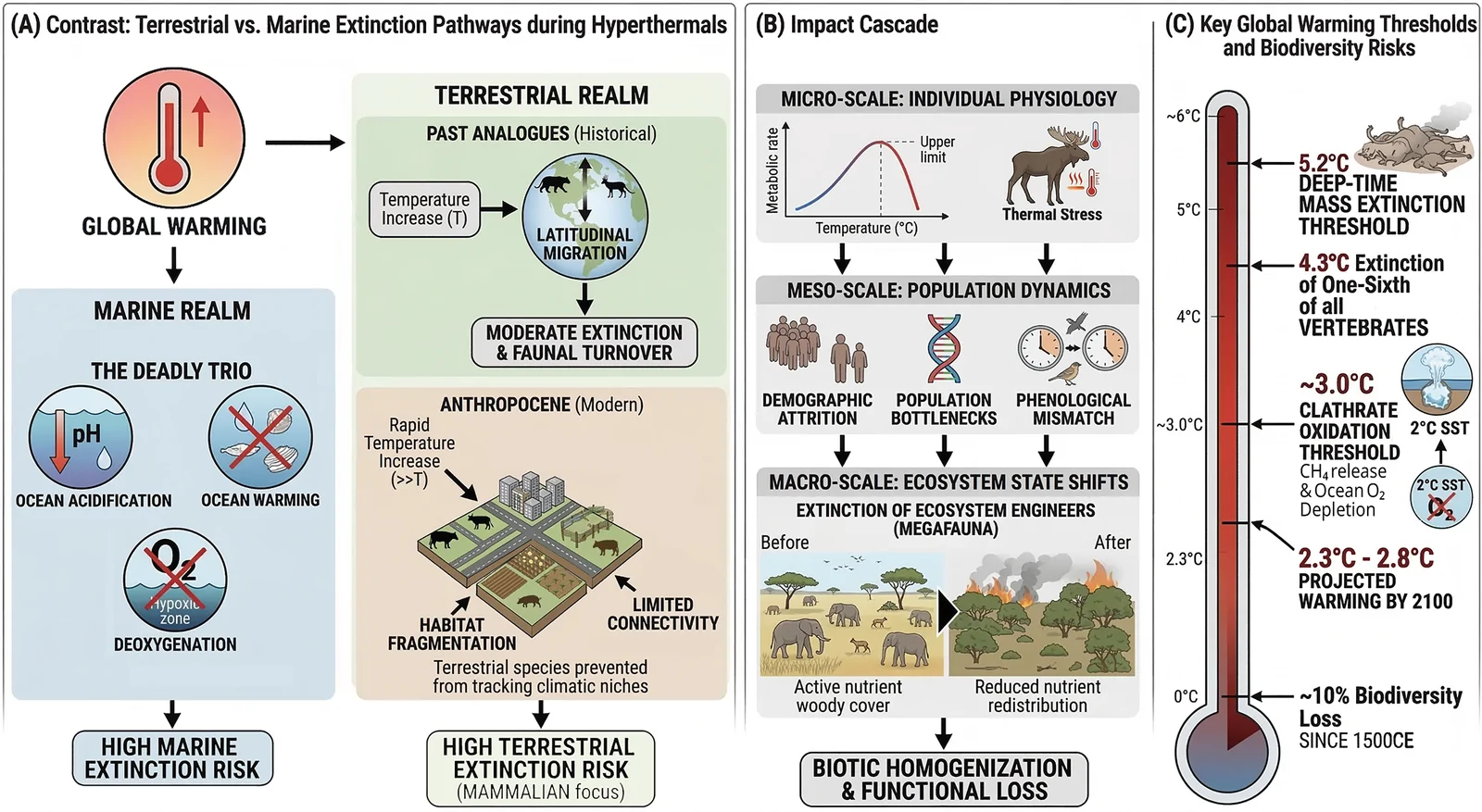

## Riassunto

L’attuale crisi della biodiversità è guidata principalmente dalle attività umane incluso il riscaldamento globale derivante dalle emissioni antropiche di gas serra, che agiscono come complessi elementi di stress ambientale. L’aumento delle temperature compromette la fisiologia degli organismi attraverso lo stress termico, generando erosione demografica e innescando cascate ecologiche che si propagano tra popolazioni ed. ecosistemi. Il record fossile offre un archivio unico per valutare queste dinamiche, documentando le risposte biologiche a un’ampia gamma di regimi climatici del passato, inclusi periodi caldi ben oltre qualsiasi condizione sperimentata nella storia umana e previsti per il prossimo secolo. Attingendo agli eventi verificatisi nel corso del Fanerozoico, sottolineiamo i meccanismi che collegano il cambiamento climatico all’estinzione nei regni marino e terrestre. Gli episodi estremi di riscaldamento del passato rivelano un ricorrente “trio letale” costituito da riscaldamento globale, acidificazione degli oceani e deossigenazione, processi che riflettono i principali driver emergenti della crisi attuale. Concentrandoci sul Cenozoico e sulle glaciazioni del Pleistocene, esaminiamo le risposte dei mammiferi alle rapide fluttuazioni climatiche e mettiamo in evidenza il contributo sinergico del genere Homo al collasso della megafauna alla fine del Quaternario. La nostra sintesi mostra che le crisi del passato forniscono indicazioni fondamentali sulle soglie di estinzione, sulle velocità del cambiamento ambientale e sulla vulnerabilità delle funzioni ecologiche durante episodi di riscaldamento. Il confronto con gli analoghi antichi suggerisce che il rischio di estinzione terrestre futuro potrebbe superare qualsiasi evento documentato nel Cenozoico, a causa dell’inusitata rapidità del riscaldamento moderno combinata con pressioni antropiche dirette come perdita e frammentazione degli habitat. Nei sistemi marini, le proiezioni relative a riscaldamento e deossigenazione indicano intensità di estinzione comparabili ai più estremi eventi ipertermali del passato. Nel complesso, l’insieme delle evidenze indica come la Terra si stia avvicinando a condizioni termiche storicamente associate ai principali episodi di estinzione.

## Impact statement

The Anthropocene is characterized by an accelerating biodiversity crisis, with extinction rates far exceeding natural background levels and early warning signs reminiscent of past mass-extinction trajectories. The magnitude and pace of disruption raise the prospect of long-lasting, potentially irreversible ecological and evolutionary consequences. To place the present crisis in a long-term context, we synthesize fossil evidence from major intervals of climatic instability, such as the Permian/Triassic catastrophic extinction, the Eocene/Oligocene transition, and the onset of Northern Hemisphere glaciation. These events reveal how rapid climate change, physiological thresholds and cascading ecosystem disruptions have repeatedly reshaped life on Earth in both marine and terrestrial realms.

However, the modern crisis differs fundamentally from ancient analogs: direct human pressure now compounds climatic stressors. On land, habitat destruction, fragmentation, exploitation and the unprecedented velocity of contemporary warming severely limit the capacity of species to track shifting climatic niches. Together, these forces elevate extinction risk at the levels observed in past natural hyperthermal events. This perspective argues for conservation strategies grounded in the long-term behavior of the Earth system. Lessons from deep time, including threshold dynamics, rates of change and the impact of functional losses, highlight the urgency of policies that reduce warming, secure ecological connectivity and curb direct anthropogenic impacts. Such approaches offer the most promising route to stabilizing biodiversity, maintaining ecosystem functioning and safeguarding the well-being of future generations.

## Introduction

The Anthropocene, widely discussed as an informal geological epoch in which humans have become the dominant driver of Earth system change (Waters et al., 2016; Urban, 2024; Wang and Yoder, 2025), is marked by an accelerating loss of biodiversity (Johnson et al., 2017; Turvey and Crees, 2019). The crisis is also visible at the genetic, biomass and population levels. Genetic diversity is rapidly eroding within many species, increasing inbreeding and reducing adaptive potential, while severe declines in biomass are driving population bottlenecks and, in extreme cases, local or global population collapse (Lino et al., 2019; Exposito-Alonso et al., 2022; Greenspoon et al., 2023; Shaw et al., 2025). These processes are compromising ecosystems functions and resilience worldwide (Moritz and Agudo, 2013; Nolan et al., 2018; Doherty et al., 2023; Lecina-Diaz et al., 2024). The severity of biodiversity deterioration is exemplified by both current and historical extinction rates which exceed the background pace by orders of magnitude across several taxonomic groups (Andermann et al., 2020; Cooke et al., 2023; Matthews et al., 2024). The scale of disruption poses the risk of long-lasting, potentially irreversible changes to the ecological and evolutionary processes underpinning biosphere stability (Barnosky et al., 2012; Ceballos and Ehrlich, 2023; Lenton et al., 2023; Pörtner et al., 2023; Rockström et al., 2024), to the extent that some scientists assert that the planet is heading toward its sixth mass extinction (Barnosky et al., 2011; Plotnick et al., 2016; Ceballos et al., 2017; Cowie et al., 2022; but see Wiens and Saban, 2025).

The primary drivers of the ongoing biodiversity decline are anthropogenic habitat destruction, fragmentation and exploitation of natural resources. Human activities further alter the Earth system by releasing massive quantities of greenhouse gases into the atmosphere, thereby increasing temperatures worldwide (Forster et al., 2026). Warming acts as an environmental stressor across all habitats and the tree of life (Seneviratne et al., 2012; Warren et al., 2018; Stroeve et al., 2025) in a complex, nonlinear way (Raftery et al., 2017; Armstrong McKay et al., 2022; Moretti et al., 2024). The core biological challenge it poses is thermal stress. As ambient temperatures approach or exceed the thermal limits tolerated by organisms (Sinervo et al., 2010; Kingsolver et al., 2013; Chevalier et al., 2024) fitness is compromised through reduced performance, demographic attrition, and heightened mortality (Addo-Bediako et al., 2000; Sunday et al., 2012; Cahill et al., 2013; Sunday et al., 2019; Lancaster and Humphreys, 2020; Clusella-Trullas et al., 2021; Hansen et al., 2023). Physiological strain can involve the disruption of vital organs (Somero, 2010), especially during climate extremes such as heatwaves and prolonged droughts (Thompson et al., 2023; Martínez-De León and Thakur, 2024; van den Bosch et al., 2025).

Thermal stress also propagates through ecological networks by altering species interactions and creating phenological mismatches (disruptions in the timing of life-cycle events among tropically linked species) which may lead to starvation, reproductive failure or population collapse (Visser and Gienapp, 2019; Roslin et al., 2021; Liu et al., 2022; Portalier et al., 2022). In biogeographical terms, species ranges are shifting and contracting globally and unevenly across taxa and environments (Ripple et al., 2015, 2019; Beyer and Manica, 2020; Lenoir et al., 2020) generating novel ecological conditions which are expected to lower biodiversity to the advantage of few winners and at the expense of many losers (McKinney and Lockwood, 1999; Holl et al., 2022). Species already living close to their thermal maxima, as is common for tropical vertebrates (Fritz et al., 2009; Ceballos et al., 2017; Kotz et al., 2025), show the lowest inherent tolerances to further climate shifts (Maclean and Wilson, 2011; Khaliq et al., 2014; Chevalier et al., 2024; Mondanaro et al., 2024). The combined effect of thermal stress and indirect ecological cascades, together with the current high velocity of climate change (García Molinos et al., 2016) and with human encroachment (Simkin et al., 2022) are driving pervasive declines in the biomass and population size of extant vertebrate species (Barnosky, 2008; Dirzo et al., 2014; Ripple et al., 2015; Greenspoon et al., 2025).

Sensitivity to thermal stress is more pronounced near the higher end of the temperature tolerance limits in most cases, as indicated by the asymmetrical shape of the thermal performance curves, which show that organism functioning declines abruptly after a certain, taxon-specific upper thermal limit (Levesque and Marshall, 2021; Arnoldi et al., 2025). This suggests global warming is likely to increase extinction risk (Buckley et al., 2022; Penn and Deutsch, 2022), a tenet that analyses of the fossil record seem to support (Finnegan et al., 2024; Malanoski et al., 2024; Kiessling et al., 2025; Zhang et al., 2025).

Given the dramatic consequences of global warming, extending beyond the biodiversity crisis and deep into public health, wealth and commodities available to humans (Lenton et al., 2023; Pörtner et al., 2023), the study of past impacts of global warming on biodiversity has become essential. Ancient moments of extreme warmth, such as the Permian/Triassic or the Paleocene/Eocene transitions are therefore deeply investigated as best analogs for the current warming trend (Burke et al., 2018; Foster et al., 2018; Kiessling et al., 2019; Tian and Benton, 2020; Kiessling et al., 2023). Unfortunately, while our understanding of past mass extinctions is heavily biased toward the marine realm, most ecological research on the effects of global change is performed on the terrestrial realm (Benton, 2003). This mismatch likely impedes our ability to predict near-future consequences of the contemporary climate crisis. This review seeks to narrow this gap, comparing directly the effect of global warming on land and in the sea and reframing this knowledge in the context of the current warming trend.

This research goal – using evidence from past global warming events to understand and anticipate biodiversity responses to ongoing climate change – falls within the discipline of conservation paleobiology (Dietl et al., 2015; Barnosky et al., 2017), which dwells on the idea that the fossil record can provide enough information, of sufficient quality, to inform future conservation strategies (Kiessling et al., 2019, 2023). While substantial progress has been made in understanding the impacts of past climate change on biodiversity, important gaps remain, particularly in the quantitative integration of phenotypic, life history and climatic data across large spatial and temporal scales. To address this gap, large, quantitatively rich databases reporting extinct species phenotypes (inclusive of life history traits) are therefore accumulating, at least for Pleistocene mammals and humans (Faurby et al., 2018; Soria et al., 2021) and starting to include explicit climatic data (Timmermann et al., 2022; Zeller et al., 2023; Mondanaro et al., 2025). For older periods, the terrestrial record can still yield important insights into the ecological consequences of past climate change, but it is too spatially and temporally heterogeneous, and too strongly affected by poor sampling and preservation biases to be equally informative. In contrast, both a rich fossil record and the invaluable geochemical signature of climate changes surrounding past biodiversity crises provide a rich, highly informative amount of data and insights to be taken from the marine realm.

Herein, we first explore the link between climate change and extinction, for both terrestrial and marine organisms, in the fossil record. We then concentrate on the Pleistocene Ice Ages to expand the review to include the effects of human appearance, exploiting the richest paleontological record of life on land. Because of our background on mammalian paleontology and anthropology, we especially focus on, but do not restrict ourselves to, evidence coming from fossil mammal studies.

### Warming, deoxygenation and extinction mechanisms in the oceans

The fossil record is a reliable archive for testing biological hypotheses of faunal change and extinction against the backdrop of rapid, intense climate change (Barnosky et al., 2012; Vermeij and Roopnarine, 2013; Orzechowski et al., 2015; Fastovich et al., 2025). Global warming, in particular, has been a recurrent factor in biodiversity crises. As case studies, we will start by illustrating what happened at the end of the Permian, in coincidence with the largest mass extinction ever (Benton, 2018), and during the early, greenhouse Earth period of the Cenozoic, in correspondence with Paleogene hyperthermals (Bowen et al., 2006). Fuller reviews specifically focusing on these events are available elsewhere and cited below. We direct the reader toward these valuable sources to delve deeper into the geochemistry, paleontology and climatology of the events.

The Permian/Triassic mass extinction, colloquially known as the ‘Great Dying’, wiped out around 80–90% of marine animal species (Stanley, 2016; Finnegan et al., 2024). Initiated by massive volcanic activity recorded in the Siberian Traps, the volcanism released vast amounts of greenhouse gases (CO₂, CH₄) and sulfur dioxide, driving atmospheric carbon dioxide up to ten times as much as today (that is ~4,000 ppm, Cui and Kump, 2015). The primary kill mechanisms were the extreme temperatures, with warming topping >10 °C increase from pre-extinction times (Penn et al., 2018; Gliwa et al., 2022), and ocean acidification and deoxygenation (oxygen-starved oceans, Hülse et al., 2021). New research indicates that the crisis was triggered by Mega El Niño events linked to the collapse of the meridional overturning circulation (Sun et al., 2024b). On land, the environmental crisis manifested through profound disruption of terrestrial ecosystems, including widespread vegetation loss, increased wildfire activity, enhanced soil erosion and major restructuring of food webs, with evidence indicating this degradation began prior to, and intensified during, the marine extinction interval (Sephton et al., 2005; Chu et al., 2020). These processes were accompanied by declines in atmospheric oxygen availability inferred from geochemical model reconstructions (Berner, 2009; Mills et al., 2023) and by a rapid release of mercury from terrestrial reservoirs (up to ~100-fold above background levels, Dal Corso et al., 2020), together exacerbating physiological stress and inducing pervasive hypoxic conditions for terrestrial organisms. The combined hypoxia and hypercapnia compressed the altitudinal ranges available to species, reducing habitat diversity (Huey and Ward, 2005). A significant body of research indicates that the terrestrial faunal collapse was driven by the temperature-dependent metabolic squeeze, where the increased heat necessitated a higher metabolic rate while the available atmospheric oxygen decreased, leading to systemic physiological breakdown (Knoll et al., 2007; Benton, 2018; Penn et al., 2018; Chu et al., 2025). Terrestrial species were affected later and less than marine taxa in terms of extinction intensity, with the wave of extinction proceeding from mid-latitudes toward the tropics (Wu et al., 2024). A similar latitudinal pattern characterized the Late Ordovician mass extinction (which was driven by a global glaciation, Finnegan et al., 2012). In contrast, the Late Devonian and Late Triassic mass extinctions were more impactful in the tropics (Reddin et al., 2019) although the latter (Davies et al., 2017), but not the former (Smart et al., 2023), experienced end-Permian like volcanism and global warming. These idiosyncrasies confirm that the feedback mechanisms between climate change, biogeography and biogeochemical cycles and the ensuing changes in extinction risk are nonlinear (Armstrong McKay et al., 2022; Kaiho, 2022; Alsulami and Petrovskii, 2023).

The most massive marine Cenozoic extinctions were driven by extreme warming during the early Paleogene hyperthermals (Lourens et al., 2005; Lauretano et al., 2015). The first, abrupt hyperthermal occurred at the Paleocene Eocene boundary (PETM) (McInerney and Wing, 2011; Jiang et al., 2025), although evidence indicates continental-scale warming preceding its onset (Secord et al., 2010). The PETM and the ensuing Eocene Thermal Maximum were characterized by a highly dynamic global warming-dominated climate system, operating across both long (>10^6^ years) and short (<10^4^ years) timescales (Lauretano et al., 2015). The PETM was short and brutal, lasting less than ~200 kyr with global temperatures rising by ~5–8 °C (Lauretano et al., 2015). Hyperthermals were initiated by intense volcanism in the North Atlantic (Gutjahr et al., 2017) and unfolded within a climate system characterized by El Niño-like variability (Abhik et al., 2025). These conditions promoted widespread ocean deoxygenation and acidification, which in turn drove a vast extinction wave among marine benthic communities, with losses estimated at approximately 30–50% of deep-sea benthic foraminiferal species (Sluijs et al., 2014; Yao et al., 2018; Haynes and Hönisch, 2020; He et al., 2023). On land, hyperthermals forced repeated dwarfing across mammalian lineages (D’Ambrosia et al., 2017), and faunal and floral turnover, but extinction intensity was comparatively modest (Gingerich, 2003; Wing et al., 2005; Bowen et al., 2006; McInerney and Wing, 2011; Secord et al., 2012; Willard et al., 2019).

Across the Phanerozoic, several other major extinction events have been linked to large injections of greenhouse gases triggering a recurring suite of kill mechanisms, often referred to as the “deadly trio” (Bijma et al., 2013) of global warming, ocean acidification and deoxygenation (*e.g.* end Triassic, Bond et al., 2023), Toarcian, during the Jurassic (Remírez et al., 2024), and Cretaceous (Owens et al., 2013; Petrizzo et al., 2025). Within this framework, the Permian/Triassic and the Paleocene-Eocene transitions stand out as cases in which rapid global warming played a central role in driving extinction and ecological reorganization at multiple levels (Secord et al., 2012; Kiessling et al., 2023; Finnegan et al., 2024; Hatfield et al., 2025), posing a warning to us as we face another biodiversity crisis (Sperling et al., 2022; Finnegan et al., 2024; Kiessling et al., 2025). Although the current warming trend is not as intense in magnitude as during these events, its rapidity is alarmingly close (Olhoff et al., 2025). Data from multiple sources demonstrate the link between the magnitude and rate of temperature change and extinction intensity throughout the Phanerozoic (Kaiho et al., 2016; Song et al., 2021; Kaiho, 2022). While both cooling and warming events have driven extinctions in the past (Harnik et al., 2012; Harper et al., 2020; Morgan et al., 2022; Weppe et al., 2023; O’Connor et al., 2024), the rapid, high-magnitude current warming is hastening chemical and physical changes in the oceans at a rate unprecedented in the paleoclimate record for the last 55 million years (IPCC, 2023) and could reach levels never experienced during the last 300 million years (Caldeira and Wickett, 2003; Hönisch et al., 2012). Major differences between current global warming and past events stand in the comparatively lower level of hydrogen sulfide in the atmosphere and less widespread ocean anoxia (Foster et al., 2018; Penn et al., 2018). However, it is important to note that anoxia is not a prerequisite for extinction, as clearly demonstrated by end Triassic global warming whose death toll was immense, but fully anoxic conditions only developed 200–250 kyr after the extinction, during the Hettangian stage (Somlyay et al., 2023). Still, although anoxia is usually associated with global warming because warm surface water holds less dissolved oxygen and favor water stratification and the development of sulfate-reducing bacteria communities, intense cooling is consistent with some major anoxic events in the recent past (Chen et al., 2022) and may have contributed to triggering the Late Ordovician mass extinction (Bartlett et al., 2018).

Overall, although multiple ocean anoxic events (OAEs) were associated with extreme marine extinction rates (Reershemius and Planavsky, 2021) terrestrial ecosystems remained comparatively resilient. This contrast underscores a key point: the mechanisms and consequences of global warming differ fundamentally between the marine and terrestrial realms, making it necessary to examine continental systems separately in the sections that follow.

### Mammals, climate thresholds and human impacts through the Cenozoic

Cenozoic mammalian responses illustrate the strong context-dependence of extinction and faunal turnover under climate forcing that is typical of continental biodiversity (Kiessling et al., 2023). In contrast with the marine realm, where warming, acidification and deoxygenation acted as potent kill mechanisms, mammals in the Cenozoic terrestrial faunas show more heterogeneous, regionally modulated responses to climate change. This heterogeneity reflects the influence of local ecological structure, dispersal barriers, species interactions and asynchronous evolutionary histories.

Cenozoic climates progressively became cooler on average (Zachos et al., 2001). The cooling trend accelerated twice. The first episode occurred at the Eocene/Oligocene transition (~34 Ma), while the second acceleration occurred with the onset of fully Ice Age conditions in the Northern Hemisphere at the beginning of the Pleistocene (2.58 Ma) or possibly slightly before (Haug et al., 2005; Bailey et al., 2013). At that time, the amplitude and intensity of climate change became more extreme, the quality of the fossil and paleoenvironmental record reached its highest (Timmermann et al., 2024) and the new biggest extinction factor, the genus *Homo*, entered the scene.

The Eocene/Oligocene cold spell was sufficiently intense, and its effects long-lasting enough to mark the transition from the greenhouse Earth to the icehouse Earth (Katz et al., 2008). On land, this climatic deterioration was associated with a profound environmental reorganization and correlates in Europe with a major faunal turnover, known as the Grande Coupure. The Coupure is best described as a massive, diachronic turnover event (Prothero, 1994; Hooker et al., 2004), which nonetheless involved sizeable extinction among mammals (de Vries et al., 2021; Weppe et al., 2023), crocodylians (Mannion et al., 2015) and plants (Jaramillo et al., 2006). While this turnover coincided with global climatic deterioration, its expression was strongly modulated by regional biogeographic contexts, and the same global climatic transition had little detectable impact on mammalian diversity in North America, underscoring the nonuniform response of terrestrial faunas to global climate change.

The diversity pattern of Cenozoic North American mammals has been suggested to covary with long-term climatic trends (Figueirido et al., 2012). Such covariation has been interpreted as consistent with the idea that large-scale faunal dynamics and the termination of stable mammalian chronofaunas may be subjected to climatic forcing over vast macroevolutionary timescales. Yet, alternative explanations for the pace of biodiversity change exist (Alroy, 2008; Bush and Payne, 2021), and the very essence of mammalian chronofaunas must be considered in the light of diachronous appearance events (Prothero et al., 1998; Lister and Sher, 2001; Raia et al., 2009). Still, the faunal compositional shifts can be mediated by ecological and biogeographic mechanisms rather than direct climatic forcing (Fortelius et al., 2014).

In Europe, the Vallesian Age of the Late Miocene (lasting from 11.1 to 8.7 Ma), witnessed a profound reorganization of mammalian communities, transitioning from the relatively stable, humid and forest-dominated environments of the Middle Miocene to the more seasonal, open and arid landscapes characteristic of the succeeding Turolian Age. While the Early Vallesian is characterized as a period of ecological mixing, where newly arrived immigrants like the equid *Hipparion* coexisted with relic Middle Miocene forest-dwelling forms such as the suid *Listriodon* and the cervid *Euprox*, the onset of higher seasonality, decreased precipitation, and intense cooling at around 9.7 Ma (Domingo et al., 2013) triggered an extinction wave affecting forest-dwelling taxa and precipitating a major faunal turnover across the continent (Agusti and Moya-Sola, 1990). Although climate change seems to be the driver, the restructuring of Vallesian ecosystems is best understood as a combination of climatic deterioration, vegetation shifts, dispersal events and the diachronous nature of mammalian chronofaunas (Casanovas-Vilar et al., 2014). The same pattern appears for different reasons during the Great American Biotic Interchange (GABI). Both the early Vallesian and GABI produced highly diverse, supersaturated communities where novel immigrants coexisted with endemic faunas for some periods (Webb, 2006; Agustí et al., 2013), until severe extinctions decimated native species, especially in South America during GABI (Agusti and Moya-Sola, 1990; Cione et al., 2015), with the contribution of climate change, the immigration of novel placental predators and human hunting (Cione et al., 2003; Faurby and Svenning, 2016). In general terms, species-level analyses suggest that global climate change is more consistently associated with increases in turnover and evolutionary rates than with large, synchronous changes in absolute diversity or extinction intensity across continents (Alroy, 2000).

The beginning of the Pleistocene was marked by a similar faunal turnover, although of lower extinction intensity. Unlike the Grande Coupure, which involved substantial extinction, early Pleistocene turnover was dominated by range shifts and taxonomic replacement rather than high extinction intensity. Extinctions driven by the Pliocene/Pleistocene cooling (Brigham-Grette et al., 2013) are recorded in Africa (Bibi and Cantalapiedra, 2023), Western Eurasia (Raia et al., 2009) and among marine mammals and other large oceanic vertebrates (Pimiento et al., 2017). The intensity of extinction during both events was lower than at the Cretaceous/Paleogene transition, and lower than the current rate (Hatfield et al., 2025). However, the pace of species loss was set to change completely at the end of the Quaternary. Approximately between 50,000 and 5,000 years ago, numerous large-bodied mammals, or the megafauna, disappeared across the globe (Brook and Bowman, 2002; Koch and Barnosky, 2006). This period has been the subject of extensive scientific debate concerning the relative roles of climate change *versus* human impact as primary drivers, and fueled by the limited availability of direct extinction date estimates (Brook and Bowman, 2002; Koch and Barnosky, 2006; Stuart and Lister, 2007; Bradshaw et al., 2021).

The role of environmental change in late Quaternary megafauna extinction is multifaceted, with evidence supporting its influence on species vulnerability and availability of suitable habitats. Extinctions often coincided with major climatic shifts, including repeated Heinrich events and other abrupt stadial phases, the Last Glacial Maximum (LGM), and the subsequent warming during the Pleistocene–Holocene transition (Lima-Ribeiro and Diniz-Filho, 2014; Cooper et al., 2015; Seersholm et al., 2020; Graipel et al., 2024). In Eurasia, the LGM created strong selection pressure that led to the disappearance of warm-adapted species, including *Palaeoloxodon antiquus* and *Hippopotamus amphibius* (Stuart and Lister, 2007; Carotenuto et al., 2016). The loss of suitable habitats due to shifting climates was a key mechanism. In South America, the replacement of cold grasslands with *Nothofagus* forests during warming periods correlated with the local extinction of open-habitat grazers such as *Hippidion* and *Lama* (Metcalf et al., 2016). Similarly, in the Great Lakes region of North America, the decline of open spruce parklands during the Younger Dryas likely contributed to the extirpation of mastodons and mammoths (Meltzer, 2020). Species responded to these changes in different ways. Many species shifted their geographic ranges to track suitable habitats, with those moving over longer distances in Eurasia tending to survive longer (Raia et al., 2012). Other populations retreated to areas with less harsh conditions, known as refugia, such as southern Europe (Sommer and Nadachowski, 2006; Stewart et al., 2010). The hypothesis of climate as the sole driver is clearly insufficient to explain end-Pleistocene extinction patterns, especially where humans acted as immigrants and met species naive to their presence (Milberg and Tyrberg, 1993; Gittleman and Gompper, 2001). Extinctions were not globally synchronous (Surovell et al., 2016) but often followed the arrival of humans on different continents, still weakening the idea of a single, global climatic trigger (Brook and Bowman, 2005; Johnson et al., 2013). The ‘overkill hypothesis’ (Martin and Leakey, 1967; Martin and Klein, 1984) posits that human hunting was in fact the primary driver of extinction, a view supported by strong chronological coincidence and selectivity, and by explicit modeling of human prey harvesting and population growth (Alroy, 2001; Fordham et al., 2022, 2024; Todorov and Alroy, 2025). For some species, synergistic effects of climate change and human overkill are reasonably supported (Nogués-Bravo et al., 2008; Wroe et al., 2013; Mondanaro et al., 2019; Meltzer, 2020), and it is plausible that the temporal sequence of climatic instability followed by human arrival repeatedly created extinction-prone conditions.

Regardless of the extinction driver, the loss of the megafauna caused profound and lasting ecological consequences, leading to significant ecological state shifts. As megaherbivores, such as mammoths and mastodons, ceased acting as ecosystem engineers – a role attached to elephants in modern ecosystems (Owen-Smith, 1988; Tambling et al., 2013) – landscapes saw an increase in woody vegetation (Owen-Smith, 2001) enhanced fire regimes (Gill, 2014), and altered predator to prey ratios, which in turn impacted the survival of smaller species *via* apparent competition (Meloro et al., 2007; Raia et al., 2007; Mondanaro et al., 2017). Through their activities, the megafauna provided key ecosystem services, including regulation of vegetation structure, maintenance of habitat heterogeneity, and long-distance nutrient redistribution (Doughty et al., 2013, 2016; Pedersen et al., 2023). By consuming large quantities of plant biomass and dispersing large-seeded fruits over long distances, megafauna facilitated both seed dispersal and gene flow while transporting nutrients across landscapes *via* dung, urine and carcasses (Malhi et al., 2016). The loss of these processes reduces ecosystem productivity, stability and resistance to environmental perturbations (Smith et al., 2010; Smith et al., 2016a; Pedersen et al., 2023; Carter and Alroy, 2025), resulting not only in taxonomic impoverishment but also in reduced functionality, and limiting the capacity of ecosystems to buffer climatic variability and recover from disturbance (Malhi et al., 2016; Smith et al., 2016b; Galetti et al., 2018; Tóth et al., 2019; Berti and Svenning, 2020; Fricke et al., 2022; Smith et al., 2022; Brook et al., 2025). These state shifts, defined as the persistent, nonlinear reorganizations of ecosystem structure and function that result in alternative stable or quasi-stable states following major disturbances (Scheffer et al., 2001; Barnosky et al., 2016), imply increased extinction risk and further biodivesrsity loss because of biotic homogenization (the increased similarity of species composition among ecological communities (McKinney and Lockwood, 1999; Fraser et al., 2022, but see Keck et al., 2025).

Besides making kills, late Pleistocene humans were themselves victims of climate change. Abrupt climatic cooling may have forced extinction in several *Homo* species, through reduced climatic niche space, fragmentation and exacerbating interspecific competition (Shea, 2008; Melchionna et al., 2018; Raia et al., 2020; Hu et al., 2023; Margari et al., 2023; Muttoni and Kent, 2024; ÓhÉigeartaigh, 2025). Even *Homo sapiens*, at once the most successful species and the only survivor of the *Homo* lineage (Gilpin et al., 2016; Zeller et al., 2023) almost vanished during the climatic changes triggered by the Toba eruption (Ambrose, 1998).

### The accelerating crisis: Paleoclimate analogies and projections

**Figure 1.**
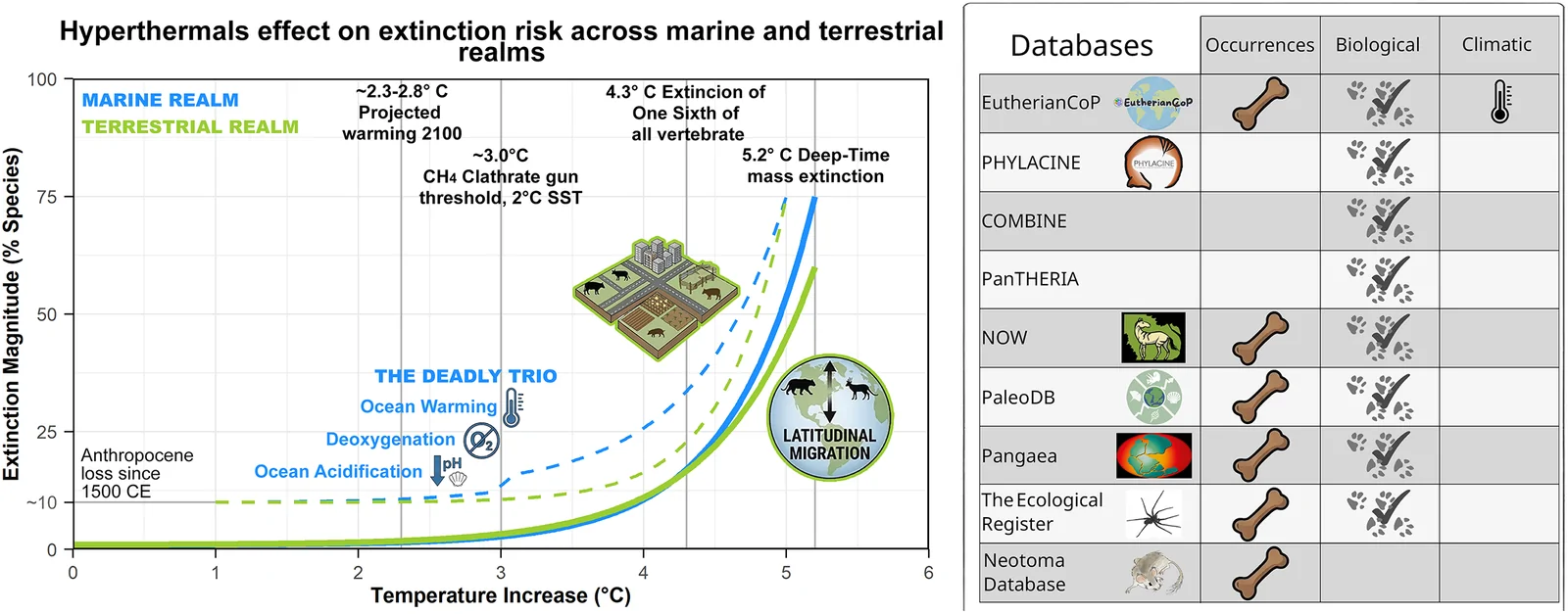
Conceptual representation of extinction dynamics under global warming. Schematic illustration of how extinction risk may respond to increasing global temperature in marine and terrestrial realms. The trajectories are conceptual and intended to highlight qualitative differences in system behavior with progressing global warming. Solid trajectories reflect the extinction magnitude *versus* warming distribution consistent with past hyperthermals event. The dashed lines represent the hypothetical trajectories tweaked by anthropogenic effects, both on lands and at the sea. In the oceans, extinction risk is shown to increase steeply with warming, reflecting the combined effects of temperature rise, deoxygenation, and acidification (the “deadly trio”) which is buffered by the ocean thermal behavior under modest warming. On land, extinction dynamics under hyperthermals are less intense than in the oceans, and more context-dependent, with risk distinctively increasing in the Anthropocene as warming interacts with human-generated stressors such as habitat loss, fragmentation and direct exploitation. The position of key temperature thresholds along the horizontal axis is based on published estimates but should be interpreted as indicative ranges rather than precise tipping points. The figure assumes relatively rapid warming trajectories comparable in their ecological consequences to hyperthermal events, implying slower rates of warming would likely lead to different outcomes. Citations for the key temperature thresholds mentioned in the figure, from left to right, refer to the 2025 Emission Gas Report (Olhoff et al., 2025), the clathrate gun hypothesis, identifying at 2 °C SST (Babila et al., 2022) the critical temperature increase triggering further warming *via* a feedback loop (roughly corresponding to 3 °C on the continents), the finding that under the worst-case (RCP 8.5) global warming trajectory, ~16.7% of species are threatened with extinction (Urban, 2015) and the average warming level (5.2 °C) triggering a mass extinction in the past (Song et al., 2021). Right: The matrix compares the data coverage of major macroecological and paleontological databases for mammals, meant to help conservation paleobiology efforts to mitigate global warming effects. From top to bottom: EutherianCoP (Mondanaro et al., 2025), PHYLACINE (Faurby et al., 2018), COMBINE (Soria et al., 2021), Pantheria (Jones et al., 2009), NOW (https://nowdatabase.org/, 2026), PaleoDB (https://paleobiodb.org, 2026), PANGAEA (Felden et al., 2023), the Ecological Register (ecoregister.org, 2026) and Neotoma (Williams et al., 2018). Figure 1. long description.

The evidence from the fossil record examined here, spanning the last 300 million years, from the early Permian through the Cenozoic and Pleistocene, provides reference information for evaluating the severe risk inherent in the current global warming trend. The first and most important lesson to learn is that since the modern biodiversity crisis is driven by direct anthropogenic pressures fragmenting, converting and reducing the extent of natural habitats and their associated biodiversity, the effective temperature increase needed to elicit a mass extinction is probably lower than inferred from deep-time global warming analogs, especially on lands (Figure 1). The initial goal of the 2015 Paris Agreement, limiting warming to 1.5 °C above pre-industrial levels, is now largely understood to be a milestone that the world is almost certain to miss (Tanaka and O’Neill, 2018; Brecha et al., 2022; Lee et al., 2023). Recent scientific analyses found that even if every nation fully implements its stated 2030 climate action plans, global warming is projected to stabilize between 2.3 °C and 2.5 °C by the end of the century, and possibly up to 2.8 °C (Olhoff et al., 2025). This means that we are off-track with the Paris agreement, and, soberly, more on track toward a scenario consistent with thermal stress thresholds identified for mass extinctions. Deep-time analyses suggest that the warming threshold to trigger a mass extinction is 5.2 °C (Song et al., 2021; Kaiho, 2022), which is higher than the present-day figures but refers to a world without human-induced disturbance on biodiversity. Anthropogenic activities likely reduce the threshold by bringing species closer to their physiological limits with little space and little time to move towards more favorable habitats. As a matter of fact, early signs of hyperthermal conditions resembling the mass extinctions’ records are already being registered. Current measurements confirm that ocean systems are experiencing fast chemical and physical changes in line with the worst hyperthermals registered over the past 300 Myr (Caldeira and Wickett, 2003; Hönisch et al., 2012). The ocean has absorbed approximately 25–30% of anthropogenic carbon dioxide, leading to a drop in average surface pH from 8.2 to approximately 8.1 (Lee et al., 2023). On the pH scale, this represents a nearly 30% increase in acidity, a level that inhibits the ability of calcifying organisms to build their shells (Peters et al., 2015; Penn and Deutsch, 2022) and is happening at a faster rate than during the PETM (Bijma et al., 2013; Haynes and Hönisch, 2020). These conditions closely resemble end-Permian and end-Triassic environments (Greene et al., 2012; Clarkson et al., 2015) and were a factor in reef species biodiversity drops over the last 500 Myr (Kiessling and Simpson, 2011). Concurrently, the oceans hold less dissolved oxygen. Coupled with nutrient runoff leading to microbial respiration, this is driving widespread deoxygenation (Stramma et al., 2008; Keeling et al., 2010) to the extent that the global oxygen inventory reduced at least 2% since the mid-20th century (Schmidtko et al., 2017). The deoxygenation is linked to high atmospheric CO_2_ levels today as it was in past (Oschlies et al., 2018; Bauer et al., 2024). The expanding hypoxic ‘dead zones’ (Diaz and Rosenberg, 2008) particularly noted in coastal areas such as the Gulf of Mexico and the Baltic Sea (Rabalais et al., 2009), mirror equally hypoxic seas associated with major marine extinctions (Falkowski et al., 2011). The recent increased uptake of dissolved nitrogen by microorganisms (Sun et al., 2024a; Beman, 2025), is itself a recurrent signature of massive extinction in the oceans, as these microorganisms deplete the oceanic nitrate, reducing ecosystem productivity and increasing toxic ammonium. The same pattern occurred during mass extinction events: end Ordovician (Finney et al., 2007), end Devonian (Liu et al., 2016) and end Permian (Knies et al., 2013; Sun et al., 2019). The combination of a warmer, more acidic, and less oxygenated ocean constitutes the most significant threat to marine biodiversity, effectively placing the oceans’ ecosystems under chemical and physiological siege (Penn and Deutsch, 2022). Finally, although the global relevance of this phenomenon is uncertain, oxidation of continental shelf clathrates can in theory transform the methane they sequester into CO_2_, consuming dissolved oxygen (Dickens et al., 1995). This positive feedback mechanism can rapidly accelerate ocean’s oxygen depletion and increase warming and acidification with as little as 2 °C increase in sea surface temperature (SST, Babila et al., 2022). This figure is equivalent to ~3 °C warming of Earth’s atmosphere, much less than Song et al.’s 5.2 °C temperature threshold.

Although current estimates of extinction rates in the marine realm are much lower than on land, the difference vanishes as data shortage is accounted for (Pimm et al., 2014; Webb and Mindel, 2015), implying defaunation is ongoing at comparable paces in both environments (McCauley et al., 2015). Future predictions are equally gloomy since marine species naturally lack the terrestrial species’ opportunity to track their preferred environments or settle within refugia, given their lower thermal tolerance and the mobility of marine habitats (Sunday et al., 2011; Hodapp et al., 2023; Albano et al., 2024).

On land, things are even more worrisome. Terrestrial wild species currently constitute 1.8% of the total biomass of mammals (the remaining part being humans plus the livestock). Marine mammals do not fare much better at 3.6% (Greenspoon et al., 2023). Furthermore, 40% of the wild mammal biomass is concentrated in just 10 species, and most of the mammalian wildlife is losing population size and lives scattered in fragmented habitats, facing heightened extinction risks (Fahrig, 2003; Haddad et al., 2015; Lino et al., 2019; Mondanaro et al., 2021; Britnell et al., 2023). The signs of climate warming-induced deterioration on extant mammal species are already evident, including miniaturization (Sheridan and Bickford, 2011; Smith et al., 2018) as during the Eocene hyperthermals (Secord et al., 2012; D’Ambrosia et al., 2017), range shifts and contraction (Sunday et al., 2012; Mitchell et al., 2018; Beyer and Manica, 2020), and physiological evidence of thermal stress (Hetem et al., 2014; Mitchell et al., 2018; de la Fuente et al., 2025), which is not even sparing the livestock (Nardone et al., 2006; Thornton et al., 2021). As with the Late Pleistocene, large mammals are especially targeted because of their vulnerability to warming (Ripple et al., 2015, 2019). Conversely, small mammals, with their high surface-area-to-volume ratio, can exchange heat with the environment more efficiently, granting them a survival advantage under thermal stress (Brook and Bowman, 2005).

Although most past warming events generally caused lower extinction rates on land than in the oceans, the current trend is, by reference, ten times faster than during PETM (Zeebe et al., 2016; Gingerich, 2019; Haynes and Hönisch, 2020), which means 1 °C in 50 years. Such a rapid pace exceeds the adaptive capacity of most terrestrial species (Opdam and Wascher, 2004; Burrows et al., 2011; Wang et al., 2023; Belfiore et al., 2024), and occurs across human-encroached landscapes, severely limiting the ability of terrestrial organisms to track suitable climates through range shifts (Hof et al., 2011; Sandel et al., 2011; Haddad et al., 2015; Urban, 2015; Lino et al., 2019; Lenoir et al., 2020). These constraints highlight the need for modern climate-change adaptation strategies grounded in physiological limits and dispersal barriers (McGuire et al., 2016; Beger et al., 2022). These peculiar, unprecedented settings make the current biodiversity crisis unique and may well imply terrestrial species are in danger more than ever before during the Cenozoic (Hatfield et al., 2022). This implies that rather than end-Permian or PETM, the current global warming is potentially more reminiscent of the K-Pg or end-Triassic mass extinctions, when the terrestrial fauna was severely hit, at least as much as, if not more than, ocean life (Cribb et al., 2023). While determining whether or not we are amidst the sixth mass extinction is a scientifically sound and important question (Barnosky et al., 2011; Ceballos et al., 2017; Wiens and Saban, 2025), studies accounting for data deficiency in poorly studied groups inform that we may have lost as much as ~10% of Earth’s biodiversity during the last 500 years (Cowie et al., 2022). The potentially devastating economic and health consequences of this biodiversity loss call for immediate, unwavering action (Lenton et al., 2023; Pörtner et al., 2023).

### The macroeconomics of climate inertia

The economic and existential costs of the global warming trajectory are staggering. Some estimates suggest it will cost as much as 12% Gross Domestic Product (GDP) for each degree of global temperature increase and hundreds of thousands of lives in the next few decades (Kemp et al., 2022; Lenton et al., 2023; Pörtner et al., 2023; Bilal Diego Känzig et al., 2024; Callahan et al., 2025). Therefore, the impact of implementing an immediate policy shift overturning the current immobility of political and economic systems – and the fallout if such a change does not occur – is central theme in macroeconomics and political economy. This inertia is not an accident, but a consequence of market and governmental systems driven by self-interest. The delay in implementing corrective climate policy is fundamentally rooted in short-termism and collective action failure (Basurto and Ostrom, 2009). Political and corporate entities operate on short cycles (elections, quarterly earnings) that prioritize immediate financial stability over the long-term, nonmonetized benefits of climate action. The global nature of climate change creates a powerful incentive for nations and corporations to ‘free-ride’, benefiting from the emissions cuts of others while bearing minimal domestic costs, leading to a collective failure to protect the shared natural resources, a typical ‘tragedy of the commons’ situation (Hardin, 1968). The flaw is then amplified by powerful actors engaging in rent-seeking (Helm, 2010), which is known to limit effective policies such as cap-and-trade programs (Rode, 2021). Rent-seeking describes the use of political and institutional influence, such as lobbying, to extract a larger share of existing wealth, rather than creating new wealth through innovation. In the context of climate policy, this manifests as the political obstruction and regulatory capture by incumbent fossil fuel interests aiming to protect the value of their vast, carbon-intensive assets against the necessary transition (MacKenzie, 2017). This persistent preference for short-term private gain over long-term public interest drives the global economy toward the high-risk, 2.5–2.8 °C trajectory.

The primary failure identified is the continued reliance on economic frameworks (e.g., GDP, standard discount rates) that treat nature as a substitutable factor of production and biodiversity loss as an incremental, linear cost (Raworth, 2018; Dasgupta, 2021; Lean, 2025). Given that humanity is rapidly eroding its natural asset base, it is increasingly necessary to recognize the natural capital as a fundamental, nonsubstitutable asset, internalizing the long-term, extinction-level costs of climate change that are currently externalized by short-term rent-seeking behaviors, thus preserving intergenerational justice (Browne, 2025). Economic discounting devalues future environmental costs, which is incompatible with the temporal scale of extinction. Contemporary ecological governance is transitioning from simplistic reforestation targets, following the scientific recalibration of mass-planting initiatives (Bastin et al., 2019; Fleischman et al., 2020), toward performance-based finance models like the Tropical Forest Forever Facility (Tropical Forest Forever Facility Secretariat, 2025). Concurrently, the 30 × 30 marine mandate is advancing through the legal framework of the High Seas Treaty, though effective protection remains hindered by substantial financing gaps (For the Ocean, 2025). Ultimately, stabilizing planetary boundaries requires internalizing environmental externalities through True Cost Accounting (Food and of the United Nations, 2023) and addressing the ethical imperative for Western nations to reduce unsustainable material consumption (United Nations Environment Programme, 2024). These and other solutions presented in the literature are increasingly synergistic (Pörtner et al., 2023) and point to the need to protect biodiversity by implementing nature-based solutions for climate mitigation that take into account the ‘natural’ cost of resources (Roe et al., 2021).

## Data Availability

No original data accompany this manuscript.

## Figure 1. Long description

Left Panel Line Graph The horizontal axis represents global temperature increase over the -normal- baseline in degrees Celsius. The vertical axis represents extinction magnitude as a percentage of species going extinct. Blue lines represent the marine realm and green lines represent the terrestrial realm. Solid lines illustrate baseline trajectories from deep-time hyperthermals. Dashed lines illustrate idealized trajectories modified by anthropogenic influences. Vertical gridlines mark several temperature benchmarks. Right Panel Database Matrix The right panel contains a table listing nine databases vertically in rows. Data availability is indicated by icons, where a bone icon represents occurrences.

Navigate back to Figure 1..
