# Supplementary material for: Warming, thresholds and extinctions: Lessons from past hyperthermals on land and at sea
Source: Camb Prism Extinct. 2026 Aug 3;4:e12. doi: 10.1017/ext.2026.10020 (PMC13748986; doi:10.1017/ext.2026.10020)
Supplement: Peer review history

# Reviewer report: Review: Warming, thresholds and extinctions: Lessons from past hyperthermals on land and at sea — R0/PR2

**DOI:** 10.1017/ext.2026.10020.pr2

Overall, this review is thorough. While typos found were mostly minor, there are several of them. I provide line-by-line feedback to that end. My biggest criticism of this review is that it does not appear to draw a novel conclusion in summarizing the literature, the authors have not made a strong case as to what is new to be gleaned from their review. They don’t provide any kind of analysis that synthesizes the literature they present. This lack of synthesis is primarily why I recommend a major revision.

Some of the literature is cited out of context. For example:

Line 98: This sentence should be revised, it implies these references are about primary drivers of biodiversity decline. However, Hansen et al. 2013; Koven et al. 2011; and Mercer 1978 are not about biodiversity decline, they are about drivers of climate change.

The rest of my feedback is primarily catching minor error, typos, inconsistencies etc. The authors should go through the manuscript and address these.

Line 119: change “as it is common” to “as is common”

Line 129: “peculiar” is perhaps not the best choice of words. The shape is not necessarily odd or unusual. Perhaps you could refer to the asymmetry of the curve.

Line 142: This is a clarity issue, beginning a new paragraph with “This research goal” it is not clear what you are referring to.

Lines 149-151: this is too broad of a statement, we certainly can, and do, understand some things about the impacts of past climate change. This statement undermines your manuscript.

Lines 164-165: what you wrote on lines 149-151 contradicts this.

Line 247: “The takeover” should be “The takeaway”

Line 271-272: the final sentence of this paragraph is an incomplete sentence.

Line 279: This is a reference that seems out of context. The placement of the reference seems to imply it is a reference addressing the quality of the fossil and paleoenvironmental record, but it is not.

Line 344: should read “late Pleistocene humans themselves fell victim to climate change”

Line 424: should read “hundreds of thousands of lives”

# Reviewer report: Review: Warming, thresholds and extinctions: Lessons from past hyperthermals on land and at sea — R0/PR3

**DOI:** 10.1017/ext.2026.10020.pr3

**Authors:** Sara Kahanamoku

**Affiliations:** University of Hawai’i at Manoa, United States

*“Extinctions and climate change: What the big fossil library tells and how far we can go with that”* reviews biodiversity trends over the Phanerozoic and highlights common drivers of major extinction events, with a special focus on climate changes that are similar to those of the contemporary climate crisis. It is clear that the authors have particular knowledge relating to Cenozoic mammalian faunas, and as a result the latter half of the paper reviews mammalian biodiversity trends over the past 65 million years, during which time several extreme and rapid warming events occurred that are potential analogs for near-term climate scenarios (e.g., as early as 2100 CE). The manuscript ends with a short overview of the economic impacts of contemporary and near-future biodiversity losses, as well as the political complexities that have made climate action difficult to achieve on a global scale.

The manuscript has much potential as a synthesis of the current knowledge surrounding long-term biodiversity patterns and the commonalities among past extinction events. In my experience, the fossil record is a powerful tool for understanding the range of possible future biodiversity outcomes under climate change, but given the complexities unique to each extinction scenario, distilling this information in a clear and succinct way can be difficult. I believe that this manuscript can be a useful and much-needed tool for communicating lessons from the fossil record with non-paleontologists, as well as will likely inspire new research questions.

However, I think that several major revisions are needed to help the manuscript reach its full potential. I suggest that the authors restructure the manuscript in order to make its central message(s) more clear. I found the most compelling points in the manuscript to be (1) thermal stress as a major kill mechanism through time and (2) the links between individual functional losses and declines in whole-ecosystem function during the Cenozoic. I have suggested several major revisions below that seek to highlight these key points, as I believe that this manuscript can make an exciting contribution to our understanding of how acute and/or rapid kill mechanisms alter individual organismal function, and in how these individual losses compound to create functional turnover within entire ecosystems and biomes.

In summary, I believe that this manuscript has the potential to serve as a much-needed synthesis of the impacts of warming on terrestrial organisms and ecosystems. Its focus on terrestrial fauna is particularly important, as our understanding of past extinctions is heavily biased towards the marine realm while our understanding of contemporary ecology is heavily biased towards terrestrial realm – a major mismatch that likely impedes our ability to predict near-future consequences of the contemporary climate crisis! My hope is that the revisions I suggest below can help strengthen the manuscript and support the authors in developing a much-needed synthesis of climate change impacts on terrestrial biodiversity and ecosystem function.

**MAJOR COMMENTS**

I realize that there may be language differences between myself and the authors (given that I am reviewing from the U.S.), so I apologize in advance if I have suggested revisions that conflict with the authors’ primary goals for this work. Below I have outlined four major areas of concern and suggested potential ways that the authors can address these concerns.

(**1) Clarify the central focus of the manuscript.** As stated above, I believe that this manuscript can be greatly strengthened if the authors choose a single central focus. In its current form, the manuscript is difficult to follow and it is unclear which message(s) the authors see as most important from their review of the paleontological literature. In my initial reading, I was unsure as to whether the primary aim was to review the climate drivers of mass extinction events across the Phanerozoic, or whether its central focus was instead on the Cenozoic mammalian extinctions and biodiversity losses during major warming events. I suggest that the authors rewrite the introduction to make their central aims more clear, and explicitly frame the aims of the review. For example, is the goal of this manuscript to encourage new questions in terrestrial paleontology, to highlight the selectivity of climate-driven extinction events, or to summarize our current knowledge about terrestrial biodiversity trends under past warmth? Is the audience paleontologists, policymakers, or both? Clarifying this in the introduction would help the flow of the story.

**(2) Include data:** While manuscript’s title suggests that it aims to synthesize the insights gained from the “large, quantitatively rich [paleontological] databases” amassed over the past few decades (also mentioned in lines 145-149 in the introduction), a quantitative treatment of these data is missing here. The authors repeatedly noted that there are several rich records of past climate, species-level phenotypes, and biodiversity that exist for terrestrial fauna that span back to the Pleistocene, yet only briefly describe these data. The manuscript would be significantly strengthened if the authors were to include summary figures that give readers a taste of what types of data exist, how climate proxies correlate (or do not) with measurements of biological traits and/or biodiversity, and what the fossil record tells us about functional losses under warming scenarios. I have included suggestions for potential figures below (under the section “suggestions for revision”).

(**2) Condense “climate as a driver of extinction,” focusing on kill mechanisms during warm events:** The first major section of the manuscript (lines 163-252) reviews major extinctions and their climate drivers. I found myself seeking additional specificity in sections – for example, on lines 181-183, where “Mega El Niño events” during warming were only briefly mentioned but could be a striking potential analog for near-future climate changes. I also found myself looking for more nuance in sections that discussed past cooling events (such as the end-Ordovician and the Carboniferous). In general, the brief treatment of past climate scenarios – which are numerous and complex and exceedingly difficult to include in a single review! – made it harder for me to understand the primary goal of this section. I found that the review of past climate changes in the section “Cenozoic Mammals, Humans, and Climate” (specifically lines 274-314) made a more compelling case for climate impacts on biodiversity, likely because the narrower focus on the Cenozoic helped provide specificity (versus taking a Phanerozoic-wide approach).

I suggest that the authors rewrite this section to explicitly focus on the “kill mechanisms” of past climate events for terrestrial fauna. This does not necessarily mean that marine fauna need be excluded, but perhaps the authors could instead highlight commonalities in kill mechanisms – e.g., habitat contraction due to heightened metabolic requirements under warming. The authors could also consider including an explicit comparison to the contemporary climate crisis, both for kill mechanisms (for example, see those discussed in Moffit et al. 2015: https://journals.plos.org/plosone/article?id=10.1371/journal.pone.0115246 and Penn and Deutsch 2022: https://www.science.org/doi/10.1126/science.abe9039) and for faunal turnover (e.g., Orzechowski and Finnegan 2021: https://doi.org/10.1017/pab.2020.43 or Albano et al. 2024: https://www.pnas.org/doi/abs/10.1073/pnas.2320687121). This is where including summary data, either in a figure (see suggestion below) or table, could help to show how differences in the directionality (warm vs. cold), magnitude (extreme vs. minor), and rate (slow vs. fast) of past climate change events created very different Earth system conditions that drove specific extinction conditions.

(**3) Expand on the impacts of warming on functional diversity:** I feel that most exciting contribution of this review is its potential ability to connect the impacts of acute and/or rapid warming to changes in functional diversity – both at an individual organismal level and across entire ecosystems and biomes. With some rewriting, this manuscript could improve our understanding of how short-term, individual-level impacts (e.g., thermal stress as a major driver of individual functional decline; currently in the introduction section on lines 101-134) connect to faunal turnover events (lines 262-328), and in turn create functional losses that can be considered ecological state shifts (lines 329-343). I would love to see the authors expand further on current insights connecting warming to organismal life history and ecological cascades, and in particular focus this review explicitly on terrestrial habitats and/or mammalian faunas over the Cenozoic.

**(4) Consider how to include macroeconomics:** The final section, “The Accelerating Crisis: Projections, Paleoclimate Analogies, and the Macroeconomic Intertia of Global Warming” (354-455), the ending paragraphs under (lines 420-455) felt out of place given the framing of the rest of the manuscript. I appreciate the authors’ desire to highlight the urgency of climate action and the political barriers that stand in the way of corrective policy – particularly frustrating given the eco-evolutionary consequences of inaction. However, this section as written felt more like an add-on than a critical component of the review. I suggest that the authors consider heavily revising this section, incorporating “lessons learned” from the fossil record on the near-future consequences of extreme warming, to make a case for climate action. This is another section in which figures could significantly help to illustrate the authors’ main points (see, e.g., this summary figure (https://www.iis-rio.org/en/publications/bending-the-curve-of-terrestrial-biodiversity-needs-an-integrated-strategy-2/) inspired by Leclere et al. 2020: https://www.nature.com/articles/s41586-020-2705-y; alternatively, Fig. 3 in Penn and Deustch 2020), but given space limitations may be difficult to include.

**Suggestions for revision:**

Below I have summarized my above comments and made a list of the major components I suggest for this revision. I recognize that many of my comments require significant effort from the authors, but feel that these changes are needed to help to bring the strengths of this piece to the forefront.

**(1) Change the title to better reflect the key message of the paper.**

The current title is a bit vague. I suggest making the title more specific and clear – perhaps along the lines of “Lessons from past extinctions for terrestrial fauna” or “Climate warming as a past and future driver of terrestrial extinctions” or similar.

**(2) Center the review on past episodes of extreme and/or rapid warmth as a driver of faunal turnover and functional losses within terrestrial ecosystems.**

As noted above, I suggest reorganizing the main text into three primary sections: (1) a brief introduction that outlines the goal and audience for the piece, (2) a section focused on kill mechanisms during warming events, and a section that reviews (3) impacts of warming on the functional diversity of mammalian faunas across the Cenozoic.

**(3) If possible, add summary figures that highlight key insights from past extinction events.**

I understand that this is a review paper and recognize the effort involved in generating appropriate review figures, so I leave this comment up to the discretion of the editor. With that being said, I believe that this manuscript could be significantly strengthened if the authors were to include several figures that capture the “big picture” trends that emerge from fossil datasets.

I suggest adding 2-3 figures that cover the following key topics:

● **Figure 1: Climate data, mammalian biodiversity, and major “kill mechanisms” during past extinction events.** This figure could illustrate how climate proxies correlate (or do not) with terrestrial and/or mammalian biodiversity through time. One option is to consider including a diversity curve alongside climate data (see, for example, Fig. 3 in Yasuhara et al. 2020: https://doi.org/10.5670/oceanog.2020.225; Fig. 1 in Elijo et al. 2022: https://www.pnas.org/doi/10.1073/pnas.2207845119, or Fig. 2 in Finnegan et al. 2024: https://www.annualreviews.org/content/journals/10.1146/annurev-marine-021723-095235). To demonstrate commonalities across kill mechanisms, the figure could also include markers of major warming events and the primary kill mechanisms of these events (e.g., deoxygenation, acidification, etc.) or an inset that illustrate kill mechanisms that are shared between key warming events (see., e.g., the Graphical Abstract for Malanoski et al. 2024: https://phys.org/news/2024-03-reveals-animals-vulnerable-extinction-due.html and https://www.science.org/doi/10.1126/science.adj5763).

● **Figure 2: Impacts of warming, from individual organismal physiology to biodiversity.** This figure could explicitly draw connections between the impacts of thermal stress on individual organisms and the macro-ecological and -evolutionary consequences of these changes to metabolic function. This figure could help to tie together the proposed section on the impacts of warming on functional diversity and connect concepts currently found on lines 101-134, lines 262-328, and lines 329-343. Potential examples from previous publications include figures in Cerini et al. 2023 (connecting individual life history changes to population collapse and extinction; https://www.nature.com/articles/s41559-023-01985-2) and Lawlor et al. 2024 (showing how warming drives several behavioral and ecological changes; https://www.nature.com/articles/s43017-024-00527-z). This figure could be purely theoretical (as in Cerini et al. 2023), or it could include empirical data on body size declines, effective population size, and biodiversity loss (e.g., from publications like Bergman et al. 2023: https://www.nature.com/articles/s41467-023-43426-5).

● **Figure 3 (?): Ecosystem functional losses resulting from megafauna extinctions.** The authors could also consider including information on changes to ecosystem function that resulted from megafauna extinctions (lines 319-351) as I think this is an exciting aspect of this review. I do not necessarily think this needs to be in a figure (or perhaps it can be wrapped into one of the suggested figures above, e.g., suggested Fig. 2), but it would help to make the case that warming can drive ecosystem functional declines by connecting biodiversity loss to its many consequences (which the authors describe quite well on lines 331-342!). This could look something like the illustrations of the “Pleistocene Park hypothesis” (e.g., Figure 1 in Windirch et al. 2022: https://doi.org/10.3389/fenvs.2022.893478).

**(4) Separate “Projections and Paleoclimate Analogies” from “Macroeconomics”**

I greatly enjoyed the section that details how contemporary climate changes are creating similar kill mechanisms to past extinction events (lines 357-415), but found it difficult to then make the jump to the following section on “macroeconomics” (lines 420-455). I suggest that this section be revised to foreground “projections and paleoclimate analogies,” as a careful treatment of the potential implications of the present day climate crisis for terrestrial biodiversity (summarizing the current literature as the authors have done here) is important. I also suggest that the authors expand on the information in this section, and include more detail on the estimates of range contractions, habitat loss, biodiversity losses, and extinction rate increase documented for contemporary land mammals and how these compare to past events. The story that emerges from contemporary data about potential future extinctions is compelling, but as written it is hard to see in the current manuscript.

I also would urge the authors to include a brief section that overviews data limitations – particularly making an explicit statement about the differences in the types of observations available for the fossil record compared to neontological data, and how these affect estimates of extinction rates and our ability to pinpoint drivers of biodiversity and functional loss (among many other things). Given the scrutiny around the “sixth mass extinction” (and the “Anthropocene” – see comment below!) I think it is important to briefly touch on the uncertainties inherent in the inferences we make from both fossil and neontological data.

**(5) Rewrite the conclusion**

The manuscript currently ends with a brief overview of the “macroeconomics of climate inertia” (lines 420-455). As noted above, I felt that this transition was a bit abrupt. Ending the manuscript with this section does not in my opinion, appropriately summarize the main strengths of the paper and may leave readers without key takeaways. I suggest rewriting the conclusion to summarize the key takeaway lessons from the fossil record for future extinction scenarios. The message about the urgency of climate action is important, but I believe will be better contextualized if the authors provide readers with a summary of what was reviewed. Once this foundation is laid out, then I suggest that the authors include some of their writing on the socioeconomic costs of biodiversity and ecosystem functional losses – as it is impactful to be able to place a monetary and human-life value on extinctions.

**MINOR COMMENTS**

In general, the manuscript would benefit from a closer revision for grammar and clarity. Given that my comments above will likely lead to some major changes, most of these line-by-line comments are intended to help clarify major points. In a few areas I have highlighted typos and/or wording differences that are repeated throughout the paper.

Title Page

Lines 3-4 (Title): I was personally unfamiliar with the term “big fossil library” prior to reading this manuscript, and suggest that any title revisions use terms that are more common to improve searchability. In addition, the current ending of the title (“and how far we can go with that”) is vague and was difficult for me to understand.

Impact Statement

Line 21: “irreversible changes to the biosphere” – irreversible over what timescales?

Lines 21-22: “We scanned the fossil record in search of relationships between

extinctions and climate change” – what fossil record(s) did you explore? What aspects of extinction did you analyze? Specificity would be helpful here.

Line 24 (as well as lines 157, 183, 211, etc.): “On lands” should read “On land”

Lines 24-25: “Human encroachment and the velocity of warming make the forecast for terrestrial faunas all but worse.” In which way is the forecast for terrestrial faunas worse? Adding specificity here would be helpful for readers to know how this paper adds to previous publications to synthesize potential future biodiversity outcomes under a range of climate change conditions.

Line 29: “that will also help saving lives and the economic well-being of future generations” – this ending phrase feels out of place without additional context. I suggest removing this phrase and ending the sentence on “protect ecosystem functioning and resilience.”

Abstract

Lines 32-34: To be a bit nitpicky here, the biodiversity crisis is not driven directly by human-released greenhouse gases – it is driven by warming resulting from greenhouse gas release (among other things). Consider combining the first two sentences of the abstract for clarity. For example, you could instead say something like: “Global warming resulting from human release of greenhouse gases over the past several centuries has driven the contemporary biodiversity crisis. Biodiversity losses have compounded due to the impact of warming as a complex environmental stressor, which compromises organismal fitness through thermal stress, the effects of which can compound into ecological cascades.”

Lines 34-36: I suggest adding a sentence between the sentence ending in “ecological cascades” and the one beginning with “We review…” that helps readers understand the utility of the fossil record. For example, this could note that the fossil record provides a wide range of past climate scenarios that capture warm states beyond those observed by humans but similar to those projected for as early as 2100 under middle- to high-emissions scenarios (e.g., Burke et al. 2018).

Line 38: Rather than saying “intense” consider instead “extreme ancient warming events”

Lines 39-40: Consider changing “which share mechanisms with the current climate crisis” to “mechanisms which are emerging as primary drivers of the current climate crisis”

Main Text

Line 80: Given that the proposal to include the Anthropocene Epoch as a formal unit in the Geologic Timescale was rejected by the International Commission on Stratigraphy in 2024, I suggest citing the Anthropocene Working Group here when you note that this is an “informal geologic epoch.” Another thing to consider here is that there are many differing opinions on the official start date of the Anthropocene -- some say 1952 (plutonium spike), others say ~1961, and yet others say the 1610 (e.g., Lewis and Maslin 2015). I suggest also citing here some publications from the ICS working group on the Anthropocene (found on their archived site: https://quaternary.stratigraphy.org/working-groups/anthropocene) which include publications and responses that overview the debate surrounding the Anthropocene as an “epoch” versus an “event.”

Line 82: Add citations to the sentence here (ends with “accelerating loss of biodiversity”) that corroborate the claim that biodiversity losses are occurring and accelerating. These can be from the IPCC (for example, IPCC AR6 WG2 from 2022: https://www.ipcc.ch/report/ar6/wg2/) or another large meta-analysis, as following sentences go into this biodiversity loss in more detail.

Lines 82-25: If possible, briefly add a sentence or two that details in what ways is the crisis manifesting at the “genetic, biomass, and population levels” – for example, loss of genetic diversity and severe biomass declines leading to population bottlenecks and/or collapse?

Line 84: “Is slashing ecosystems functioning and resilience worldwide” is a bit intense and not particularly descriptive; consider instead “is compromising ecosystem functioning and resilience worldwide”?

Lines 96-99: Once again I am being picky here, but greenhouse gas emission is not directly driving biodiversity loss; rather, the global-scale warming resulting from greenhouse gas emission is the driver (along with other major human impacts such as habitat modification via land-use change and overharvesting). I also suggest adding additional references to this sentence that reflect the large amount of literature published on the impacts of warming on habitat availability,

Line 99: Consider removing “global” here to have the new sentence read “Warming acts as an environmental stressor…”

Lines 145-149: This is the first time that phenotypic traits and life history are mentioned in the manuscript. As noted above, rewriting the introduction to better lead up to this sentence (which contains, in my opinion, the meat of the story!) would better set the stage for the review.

Lines 157-159: While I appreciated the context that the authors are mammalian paleontologists and anthropologists, I do not think that this is the primary reason that a review centered on terrestrial faunas and mammals is important! I suggest that the authors remove this sentence and instead include language in the introduction that make the case as to why we need a review that synthesizes the current knowledge base for mammals.

Line 176: “Which left trace in today Siberian Traps” is likely a typo

Lines 183-184: “Intense warming combined with plunged oxygen levels” – how do we know that oxygen levels decreased on land? It would help to cite references here and also include more specifics (e.g., how much did oxygen decrease?). Consider also changing “plunged” to “declines in oxygen.”

Lines 201-202: “The most important Cenozoic warming events are the Paleogene hyperthermals” – important in what way? I suggest avoiding value statements like “important” without specificity – perhaps instead say “The largest extinctions during the Cenozoic were driven by extreme warming during the early Paleogene hyperthermals.”

Line 204: “Although an earlier warming tendency was suggested” – this wording is confusing to me and I suggest rewriting the sentence for clarity.

Line 206: “Over geologically brief intervals” – how brief is “geologically brief”? Decades? Centuries? Including the rate of warming will help contextualize this event compared to the present.

Lines 207-210: This sentence as written may be read as suggesting that El-Niño events drove volcanism. I believe there may be a typo here that caused my confusion!

Lines 212-213: “Comparatively little extinction” – compared to which events? Please provide an estimate of extinction rate and/or biodiversity losses for the Paleogene and contextualize with a comparison to values for earlier periods.

Lines 216-217: “The similarity between the Permian/Triassic and the Paleocene/Eocene events is striking” – I found myself wanting the comparison between these events to be laid out before being told that they are “strikingly” similar, as I feel that the story is more compelling when value judgements follow case studies and/or data. Consider moving this sentence down and beginning the paragraph in a different way.

Lines 225-226: This sentence is a bit confusing as written. I suggest rewriting to instead say “While past events have very different causes than the current biodiversity crisis, all share similar mechanisms – and thus serve as a warning for potential future outcomes”

Line 247: “The takeover is that” – I believe this phrase may be a typo?

Line 252: Change “on lands” to “on land”

Lines 262-264: As written, the sentence implies that the correlation between Cenozoic north American mammal diversity and the benthic stack was previously thought to be real, but is no longer. Is this the case? If not, change the wording “was said to correlate with” (line 253) to reflect this. If not, consider removing and/or adding more context. This is also where a summary figure (see “major comments” above) could help.

Line 264: “This quantitative correspondence” – is this correlation quantitative? If yes, using language that reflects statistical significance would be helpful for readers; as noted in previous comment, a figure showing the data would be even more helpful!

Lines 271-273: This is an incomplete sentence and may be a result of a typo.

Lines 288-292: How much smaller in size was the faunal turnover at the beginning of the Pleistocene? As with all claims made in this paper, having numbers to give readers a quantitative sense of the magnitude of extinction/turnover would be very helpful (particularly for readers who, like me, may not be mammalian paleontologists!).

Lines 293-398: This section detailing humans as a potential driver of megafauna extinctions is very interesting particularly given that Hominids represent a potential kill mechanism that is quite unique when compared to past extinction events. It may help the flow of the paper to separate this into its own subsection by adding a header.

Lines 319-320: “Stands on thin ice” is a bit colloquial; I suggest rephrasing here.

Lines 329-332: I found this section very interesting and suggest that the authors expand further on the concept of functional losses resulting from climate change. Consider adding a few additional sentences that discuss the role of megafauna as ecosystem engineers – including both what their presence provides in terms of ecosystem services and how the loss of megafauna leads to reduction in services (detailed on lines 332-340).

Lines 335-336: How did megafauna loss reduce the diffusion of nutrients? Some additional explanation and/or a clarifying sentence here would be helpful.

Line 340: “Ecosystems state shifts” – given that opinions widely differ on what should be considered a state shift, please define what you mean by “state shift” here and cite a reference for which definition you prefer to use.

Lines 344-351: This paragraph on human extinctions resulting from climate change feels a bit out of place; however, I appreciate the way that briefly detailing how human extinctions were driven by climate change makes it clear that humans are, too, part of the terrestrial ecosystems that are at risk.

Line 357: “The evidence base from 300 million years of extinction” – what evidence base? This is where incorporating figures and/or previously published data would be helpful. I am also confused as to what timescales we are spanning here – are we discussing the Phanerozoic (thus more than 540 million years), or beginning with the evolution of terrestrial fauna (where I believe the “300 million year” estimate comes from), or focusing on the Cenozoic (65 million)? Perhaps consider rewriting this sentence to say instead “The evidence base from the fossil record…”

Lines 357-358: I am unsure what “contextually assess” means here.

Lines 366-368: This sentence could use additional clarification – for which group(s) were these thermal thresholds estimated? And how does human disturbance alter these thresholds (for example, does it lower or raise them)?

Lines 369-371: Changes to ocean systems “in line with the worst hyperthermals” – which hyperthermals? Specificity would be helpful here (as Paleozoic and Mesozoic hyperthermals are very different than Cenozoic hyperthermals), as would including an estimate of contemporary warming and how it compares to past analogs.

Lines 369-395: I found the switch from talking about terrestrial ecosystems to these several paragraphs on marine ecosystems to be a bit jarring. Perhaps it would be helpful to contextualize this section a bit more by including a sentence or two about how most of what we know about past extinctions comes from the marine fossil record – and so the ocean often is our first point of comparison when linking paleo data to contemporary records.

Lines 409-413: I am unsure what the phrases “warming did not signify extinction rate as important as in the oceans in the past” and “in a world that is hostile to habitat tracking, especially on lands” mean. Please rewrite for clarity and consider splitting this into two sentences.

# Editor report: Recommendation: Warming, thresholds and extinctions: Lessons from past hyperthermals on land and at sea — R0/PR4

**DOI:** 10.1017/ext.2026.10020.pr4

**Affiliations:** Center for International Collaboration and Advanced Studies in Primatology, Kyoto University, Japan

This manuscript was reviewed by two experts on biodiversity dynamics in the fossil record. Both reviewers praise the depth of the review, which draws from a wide range of latest studies on the impact of climate change on biodiversity, with emphasis on past hyperthermal events. At the same time, both reviewers note that original synthetic insights of this review should be clarified and underscored to make the paper more impactful--an assessment with which I agree, especially given the plethora of recently published reviews in conservation paleobiology (e.g., Finnegan et al. (2024) on marine extinctions). A conceptual figure visualizing such insights (see also suggestions by Reviewer 2) would enhance the impact.

My impression is that, with revision, this review could become an excellent follow-up to the opinion paper by Kiessling et al. (2023 [cited in this review]), which highlighted context dependence of extinctions during hyperthermal events (Fig. 1). To that end, I suggest that the authors place greater emphasis on contrasting ecological and evolutionary (including extinction) consequences of hyperthermal events in the absence versus presence of anthropogenic pressures. Indeed, it is important to acknowledge that, contrary to the authors‘ statement on Lines 32-33 & 96-97, direct habitat destruction and exploitation by humans constitute even greater threats to modern biodiversity than climate change, at least in the near term (see e.g., IUCN Red List for a global assessment; Struebig et al. (2025) [https://doi.org/10.1038/s44358-025-00064-7] for a regional review; and Bergman et al. (2023) [https://doi.org/10.1038/s41467-023-43426-5] on the causes of mammal extinctions in the ’near past'). There is, of course, no denying that the ongoing climate change is already affecting species and ecosystems, and that it raises grave concerns about the future of biodiversity, but that cannot be discussed in isolation from the concurrent and more direct anthropogenic disturbances, in the context of the Anthropocene. A lot of the relevant information is already in the manuscript, and the authors do touch on the issue (e.g., Lines 367-368), so I think what is needed in revision is repackaging of the information and sharpening of the focus, as the reviewers also suggest.

The last section on the need for macroeconomic structural shifts offers a welcome perspective that fits the interdisciplinary aim of the journal. On that note, I would be curious to read the authors' take on emerging global initiatives such as the Tropical Forest Forever Facility. In addition to the discussion of climate-change mitigation, a discussion of climate-change adaptation (see e.g., McGuire et al. 2016 [https://doi.org/10.1073/pnas.1602817113]) from the paleontological perspective may be warranted.

In revising the manuscript, I encourage the authors to carefully consider the many valuable suggestions by the reviewers, as well as the fundamental points I noted above.

# Editor report: Decision: Warming, thresholds and extinctions: Lessons from past hyperthermals on land and at sea — R0/PR5

**DOI:** 10.1017/ext.2026.10020.pr5

**Affiliations:** School of Natural Sciences, Macquarie University, Australia

No accompanying comment.

# Reviewer report: Review: Warming, thresholds and extinctions: Lessons from past hyperthermals on land and at sea — R1/PR7

**DOI:** 10.1017/ext.2026.10020.pr7

This is my second revision of this manuscript. I am largely satisfied with the revisions made in response to both reviewers. I have some line-by line edits to address typos, potentially confusing text, and some minor oversights that can be easily addressed.

Line 41: Abstract, missing a word, should read “driven primarily by human activities”

Line 123: please include a reference quanitifying the release of greenhouse gasses

Line 154: unusual use of “upper hand” do you mean at the higher end?

Line 169: what is the cited evidence for this mismatch?

Line 186“ remove ”much"

Line 209: “direct” the reader towards

Line 268: you mention these kill mechanisms several paragraphs earlier, perhaps introduce this term sooner.

Line 380: somewhere in this paragraph or the next, it would be wise to acknowledge how little data (direct dates) there actually are informing the extinction chronologies for the impacted species. The supplementals in Koch and Barnosky 2006 provide the state of knowledge at the time, but there’s only been modest improvements to that end. We seek to explain a pattern without a refined understanding of what the pattern looks like.

Line 405: this overstates what we know about extinction chronologies. Elsewhere you describe continental variation in climate impacts, but we lack detailed regional chronologies for most species.

# Editor report: Recommendation: Warming, thresholds and extinctions: Lessons from past hyperthermals on land and at sea — R1/PR8

**DOI:** 10.1017/ext.2026.10020.pr8

**Affiliations:** Center for International Collaboration and Advanced Studies in Primatology, Kyoto University, Japan

The revised manuscript was reviewed by one of the original reviewers and myself. It extensively incorporates suggestions from the original reviewers and editors. Both Reviewer 1 and I are largely satisfied by the revision and appreciate the authors' substantial efforts. I found the revised manuscript engaging, timely, and informative.

At this point, my main comments for further revision relate to the new Figure 1:

1) This figure is described as a conceptual figure, but the extinction trajectories are quantitatively quite specific. Please clarify in the figure caption (i) to what extent the lines are informed by empirical data, citing relevant sources; and (ii) what aspects of this figure are hypothetical.

2) The vertical axis should be labeled “Extinction magnitude” (see Barnosky et al. 2011).

3) I don’t understand why the stepwise increase for the Anthropocene is only applicable to the terrestrial curve above ~3.8°C. We already live in the ‘Anthropocene’ and are at about 1°C above the 1850-1900 AD baseline, but that (i.e, the Present) would fall on the green “Historical” line in the figure. Please explain or revise as necessary.

4) The horizontal axis represents the magnitude of temperature increase, but isn’t it necessary to consider the speed of change (especially relative to the range of generation time in mammals) in order to adequately predict extinctions?

5) I don’t see a strong logical connection between the right and left components of this figure, and so I would recommend turning the list of databases into a table with links (I don’t think it needs to be a figure), and identify any major differences in their data coverage in terms of occurrence records and biological information. Also, consider adding the Neotoma Paleoecology Database (https://www.neotomadb.org/).

The following are minor editorial comments and suggestions. Please also follow the detailed recommendations by Reviewer 1.

L62: It should be either “comparable to” or “likely less severe than”, but not both; please choose one.

L102: “dominant driver Earth” -> “dominant driver of Earth”

L117: “traversing its sixth mass extinction” -> perhaps “headed toward its sixth mass extinction” because we have not yet entered it (cf. Barnosky et al. 2011).

L159: “a tenet that the analysis of the fossil record seems to support” -> “a tenet that analyses of the fossil record seem to support”, since multiple analyses are cited here.

L167-168: “past extinctions” -> perhaps “past mass extinctions”? In either case, please provide some references to support this statement.

L171: “near this gap” -> perhaps “narrow this gap”

L174 and elsewhere: Please use an em-dash instead of a hyphen, where appropriate.

L195: “do not restrain ourselves to” -> “do not restrict ourselves to”

L201: Do you really mean “theories”, or are they perhaps hypotheses?

L212: “96% of standing marine diversity” -> “around 80 to 90 % of marine animal species”; see Finnegan et al. (2024:316) and e.g., Stanley (2016; https://doi.org/10.1073/pnas.1613094113); the cited review article by Benton & Twitchett (2003) actually discusses some issues with the 96% figure in Box 1.

L213: “recorded in today Siberian Traps” -> “recorded in the Siberian Traps”

L229-230: “ranges available” -> Clarify if this is referring to habitats, geographic ranges, or perhaps bathymetric ranges.

L274: “...) posing a warning to the current biodiversity crisis” -> perhaps “...), posing a warning to us as we face another biodiversity crisis” [the warning is directed to us humans and not the crisis]

L287-288: “important to notice” -> “important to note that”

L294: “contributed triggering” -> “contributed to triggering”

L313-314: “The cooling trend is punctuated by two accelerations.” -> “The cooling trend accelerated twice.”

L314-315: Rephrase as: “The first episode occurred at the Eocene/Oligocene transition (~34 Ma), and the second with the onset of fully Ice Age conditions...”

L328: “turnover coincides with” -> “turnover coincided with”

L329-330: “the same climatic transition” -> “the same global climatic transition”

L338: Add a comma after the closing parenthesis.

L340-341: “the climate change – faunal composition concordance” -> Perhaps simply “faunal compositional shifts”?

L362: “species-level data analysis suggests” -> “species-level analyses suggest”

L409: “well possible” -> “plausible”

L417: “ratios which” -> “ratios, which”

L419: “megafauna” -> “the megafauna”

L430: “ecosystems state shifts” -> “ecosystem state shifts”

L443: “by Toba eruption” -> “by the Toba eruption”

L459: “commitment” -> perhaps “goal”

L461: “Current scientific analyses” -> “Recent scientific analyses”

L466: “suggest the warming” -> “suggest that the warming”

L467: “refer to” -> “refers to”

L480: “a factor to reef species biodiversity drops” -> “a factor in reef species diversity drops”

L499: Add a comma after “CO2”, and use a subscript for 2.

L501: “SST” -> “sea surface temperature”

L506: Edit as: “...), implying ocean defaunation is ongoing at comparable paces in both environments”

L508: “naturally miss” -> “naturally lack”

L509: What do you mean by “mobility of marine habitats”? Consider rephrasing to clarify.

L516: “extinction risk” -> “extinction risks”

L521, L530: Add a comma after the closing parenthesis.

L554, L566: “Left/Right Panel” -> “Left/Right” [Fig. 1 as a whole is a panel of figures]

L554: “biplot” -> “plot”

L563: “x-axis” -> “horizontal axis”

L577: “suggesting” -> “suggest”

L580-581: “failure ... is a central question” sounds odd. Please reword.

L602: “running down its own assets” -> Please reword.

L606: “costs, that is” -> “costs, which is”

L618: “point to protect biodiversity” -> “point to [the need to?] protect biodiversity”

# Editor report: Decision: Warming, thresholds and extinctions: Lessons from past hyperthermals on land and at sea — R1/PR9

**DOI:** 10.1017/ext.2026.10020.pr9

**Affiliations:** School of Natural Sciences, Macquarie University, Australia

No accompanying comment.

# Editor report: Recommendation: Warming, thresholds and extinctions: Lessons from past hyperthermals on land and at sea — R2/PR11

**DOI:** 10.1017/ext.2026.10020.pr11

**Affiliations:** Center for International Collaboration and Advanced Studies in Primatology, Kyoto University, Japan

I thank the authors for making further edits to the manuscript. However, regarding Figure 1, my earlier comments 1(ii), 3, and 4 on the R1 manuscript are not sufficiently addressed, and so I request the authors to consider these points again:

1(ii): Although the figure nicely illustrates important qualitative aspects of extinction magnitude in response to hyperthermal events, I am concerned about the appearance of precision of the “conceptual” trajectories. For example, there is no empirical evidence that a warming of 3°C would wipe out all marine life, as depicted by the blue line. After all, the authors cite a 5.2°C threshold for a mass extinction, but the blue line reaches the mass extinction level (i.e., 75% [Barnosky et al. 2011]) at about 2.7°C, for no clear reason. Similarly, the purported ~10% loss of species since 1500CE does not appear to be reflected in the green curve for the terrestrial realm at the current state of circa +1°C above the pre-Industrial baseline, but no explanation is provided as to why. Moreover, the 4.3°C threshold is for extinction of 1/6 of vertebrates, but how that translates to the position of the red line at 75% extinction is unclear.

Given these issues, I think the authors need to: (A) clearly explain how the curves were drawn (including the evidence used); (B) explicitly state that the curves are largely hypothetical; or (C) depict the ranges of uncertainty. I also argue against extending the curves to the 100% extinction level (since we simply don’t know what it takes to completely wipe out all marine or all terrestrial life).

3: The authors state in the Response that they “have revised figure 1 according to [my] suggestions”, but I don’t see any change to the figure itself on this matter. I am still unclear as to why the step of the terrestrial curve is positioned at ~3.8°C and not at a lower level of temperature increase. If the point is to illustrate the Anthropocene synergy with human impacts, the entire green curve should be elevated. I also don’t see why there shouldn’t be additional steps (e.g., at the 3°C threshold where a positive feedback loop is said to kick in).

4: My comment was about the rate of change of temperature--For example, wouldn’t a 3°C increase have different extinction consequences depending on whether it happens over 500 years or 50,000 years? How is that aspect of warming reflected in this figure? Consider stating implicit assumptions about durations of hyperthermals or depicting different trajectories to illustrate the effect of the rate of temperature change.

The following are minor issues concerning word choice, punctuation, etc.:

L109: “compromising ecosystems functioning” -> “compromising ecosystem functions”

L153: Remove “I”.

L171: “This review is sought to” -> “This review seeks to”

L213-215: The Author Response says that the authors “have ... cited Finnegan et al. (2024) and Stanley (2016) instead of Benton & Twitchett (2003)”, but Stanley (2016) is not cited.

L221: “instigated” -> perhaps “triggered”

L241: “at the tropics” -> “in the tropics”

L263: “, faunal and floral” -> “and faunal and floral”

L276: “posing” -> “, posing”

L286: Remove extra parentheses surrounding IPCC.

L302: “motivating the need to examine” -> “motivating us to examine” or “making it necessary to examine”

L316-317: Use the past tense.

L348: “successive, Turolian Age” -> “succeeding Turolian Age”

L350: “like Hipparion” -> “like the equid Hipparion”

L364: “analyses suggests” -> “analyses suggest”

L378: Insert a comma after “years ago”.

L412: “proven” -> “supported”

L498: “patter” -> “pattern”

L499: Insert “and” before “end Permian”.

L505: Remove “that”.

L511: Insert a comma before “implying”.

L518: Remove “Worse than that”. [No need to declare which is worse.]

L520: “fragmented populations” -> “fragmented habitats”

L538: Insert a comma before “severely limiting”.

L547: “as much if not more than ocean life” -> “as much as, if not more than, ocean life”

L548: Remove “understanding”.

L563: “faunas mitigated thermal stress and moderate warming” -> perhaps “faunas mitigated thermal stress from moderate warming”?

L574: “business-as-usual” -> “worst-case (RCP 8.5)”

L597-599: Please rephrase this sentence. Neither “implementing” nor “fallout” is a “question”; a question should be in the form of how/why/what/when/where.

L599: “oversight” -> perhaps “accident”?

L601: Remove “the concepts of”.

L636: “point to protect biodiversity” -> “point to the need to protect biodiversity”

# Editor report: Decision: Warming, thresholds and extinctions: Lessons from past hyperthermals on land and at sea — R2/PR12

**DOI:** 10.1017/ext.2026.10020.pr12

**Affiliations:** School of Natural Sciences, Macquarie University, Australia

No accompanying comment.

# Editor report: Recommendation: Warming, thresholds and extinctions: Lessons from past hyperthermals on land and at sea — R3/PR14

**DOI:** 10.1017/ext.2026.10020.pr14

**Affiliations:** Center for International Collaboration and Advanced Studies in Primatology, Kyoto University, Japan

The revised Fig. 1 is easier to understand, and sufficient caveats are provided in the figure caption such that the reader is much less likely to misinterpret the trajectories depicted here.

I believe this manuscript is now ready to be accepted for publication. I really appreciate the integrative perspective of this review paper, and think that it will serve as a valuable reference and inspiration for scientists and other professionals working toward biodiversity protection under climate change.

When you submit the manuscript for copy editing, please make the following corrections:

L567: “tweaked” -> “elevated” [the shift is more substantial than a tweak]

L572: “the oceans thermal behavior” -> "the ocean’s thermal behavior"

L580-582: “position of key temperature thresholds ... is based” -> “positions of key temperature thresholds ... are based”

L609-610: “is central themein” -> “is a central theme in”

# Editor report: Decision: Warming, thresholds and extinctions: Lessons from past hyperthermals on land and at sea — R3/PR15

**DOI:** 10.1017/ext.2026.10020.pr15

**Affiliations:** School of Natural Sciences, Macquarie University, Australia

No accompanying comment.
